# Pt nanohelices with highly ordered horizontal pore channels as enhanced photothermal materials†
†Electronic supplementary information (ESI) available: XPS and TEM of the Pt nanohelices. See DOI: 10.1039/c5sc01686j


**DOI:** 10.1039/c5sc01686j

**Published:** 2015-07-23

**Authors:** Shuyan Song, Xiao Wang, Sheling Li, Zhuo Wang, Qi Zhu, Hongjie Zhang

**Affiliations:** a State Key Laboratory of Rare Earth Resource Utilization , Changchun Institute of Applied Chemistry , Chinese Academy of Sciences , Changchun 130022 , P. R. China . Email: hongjie@ciac.ac.cn; b School of Material Science and Engineering , Changchun University of Science and Technology , Changchun , 130022 , P. R. China

## Abstract

Recent studies have further demonstrated that the conjugation of noble metal helical nanostructures could alter their optical and catalytic activities. However, the intrinsic isotropic crystal growth of Pt makes the synthesis of high-quality Pt NCs with unique porous or branched nanostructures difficult. In this work, a new, powerful capping agent, *N*,*N*-dimethyloctadecylammonium bromide acetate sodium, was synthesized and used to coordinate Pt ions, slowing down the reaction rate. As a result, in aqueous solution, Pt nanohelices with highly ordered horizontal pore channels were successfully fabricated. Importantly, the Pt nanohelices were composed of several sub-2 nm Pt nanowires coiled together around a central point. The as-obtained samples exhibited enhanced photothermal properties compared with the classic Pt nanoparticles.

## Abstract 2

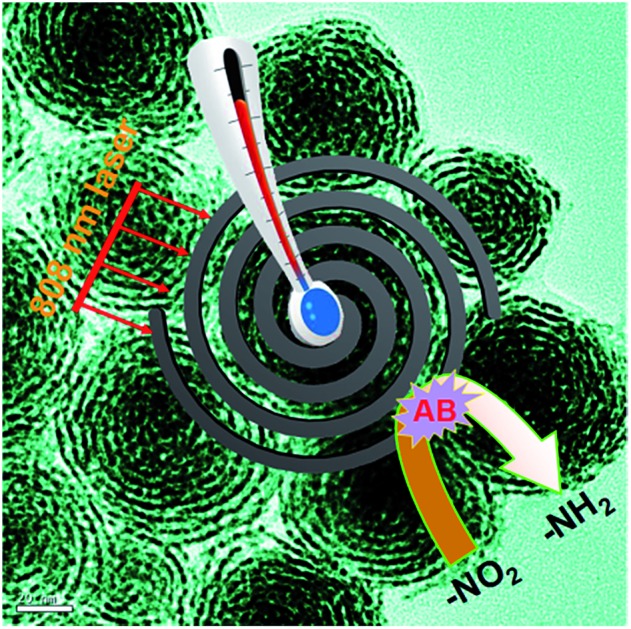
Pt nanohelices with highly ordered horizontal pore channels have been successfully developed. A study of the formation mechanism has shown that a typical two-step growth process occurs. The as-obtained Pt nanohelices exhibit enhanced photothermal and catalytic properties.

## Introduction

Chirality is widely expressed in nature, spanning from organisms to inorganic species and from the nanoscale to macroscopic scale, in which the helix is a central structural motif because of its significance in biological processes and biological evolution, as well as its versatile applications in materials science, asymmetric synthesis, sensing, and chiral devices.^1^–^6^ Constructing a helix within inorganic nanomaterials can potentially generate some unique properties that may be beneficial for their utilization beyond the traditional fields.^7^,^8^ During the past few years, considerable attention has focused on the design of supramolecular self-assembled helices based on noncovalent interactions.^9^–^14^ However, there are only a few cases of helical metallic nanocrystals in the literature due to the intrinsic lattice symmetry of the atomic packing modes. In recent years, metallic nanocrystals have begun to attract attention from both theoretical and experimental communities.^15^,^16^

Pt-based nanocrystals have received continuous attention because of their unique chemical and physical properties; such materials have been used in a wide range of applications.^17^–^21^ It is important to study the morphology-controlled synthesis of Pt nanostructures because the chemical and physical properties of Pt nanostructures can be highly influenced by the synthesis. With the development of nanoscale synthetic technologies, Pt nanocrystals with controllable shapes and sizes, including nanoparticles,^22^ nanocubes,^23^ nanowires,^24^ nanotubes,^25^ nanobranches^26^ and porous and hollow nanostructures,^27^ have been prepared. These successful examples have shown that controlling the noble metal's overgrowth process is a key factor in optimizing the morphology. A widely accepted view point is that the surface capping agent plays a significant role in the overgrowth process. It can coordinate with noble metal ions, thus the growth-rate and growth-direction can be affected strongly. Some kinds of unusual noble metal nanostructures have been successfully created by controlling the overgrowth process. As typical examples, Pt–Co nanowire assembles,^28^ Pt–Pd cube hybrids,^29^ Pt on Au nanorods^30^, *etc.* have been reported. However, to our best knowledge, there are no reports focusing on the synthesis of Pt helical nanostructures in the form of nanowires. This is in contrast to the large number of achiral metallic nanowires reported. For instance, Xia's group has reported an oleylamine-assisted method that can be used to synthesize ultrathin Au nanowires in solution.^31^ Additionally, active Au–Ag alloy nanowires containing icosahedral moieties^12^ and Au nanowires^32^ by top-down etching in ultrahigh vacuum have also been successfully constructed by José-Yacamán’s and Kondo's groups respectively. In contrast to Au nanowires, the Pt crystal structure favors isotropic growth. As a result, Pt nanocrystals often exist as small nanoparticles and it is hard to obtain ordered Pt nanowires or branched nanostructures.^33^,^34^ Therefore, it is a challenge to create novel and complex Pt helical nanostructures by assembling Pt ultra-thin nanowires. The fabrication of these helices opens the door to optical uses, which warrants further study of the cluster stability and electronic properties. Moreover, these structures are rich in edge and corner atoms that endow them with high catalytic performances.^35^–^38^

In the literature, it is concluded that the key point for inducing morphology transformations in Pt nanocrystals is slowing down their growth rate. Considering the high oxidation potential of Pt metal salts, a mild reducing agent, ascorbic acid, and a new powerful shape-directing agent, *N*,*N*-dimethyloctadecylammonium bromide acetate sodium (OTAB–Na), have been introduced in this work. As two of the most commonly used surfactants, CTAB (hexadecyltrimethylammonium bromide) and CTAC (hexadecyltrimethylammonium chloride) molecules have exhibited outstanding induction effects for the growth of noble metal particles due to the formation of CTAB or CTAC–noble metal ion complexes.^39^,^40^ However, the interactions are often not sufficiently strong to accomplish well-controlled syntheses. Introduction of a –COO– group to the quaternary ammonium salt may completely change the coordination environments of the noble metal ions, and the strongly bonded –COO–Pt precursors could significantly decrease the growth rate of the Pt nanoparticles during the reduction process. Consequently, Pt nanohelices with highly ordered helical nanostructures and horizontal pore channels could be obtained.

## Results and discussion

In a typical synthesis of Pt nanohelices, a certain amount of OTAB–Na powder and 10 mL of water were mixed and heated to 60 °C until a clear, transparent solution was obtained. Then, H_2_PtCl_6_ aqueous solution and freshly prepared ascorbic acid (AA) aqueous solution were quickly added in sequence with vigorous stirring. The resulting mixture was held at 60 °C for 2 hours. Finally, the colloidal products were separated from the reaction solution by centrifugation and washed with DMF and ethanol three times.

**Fig. 1:**
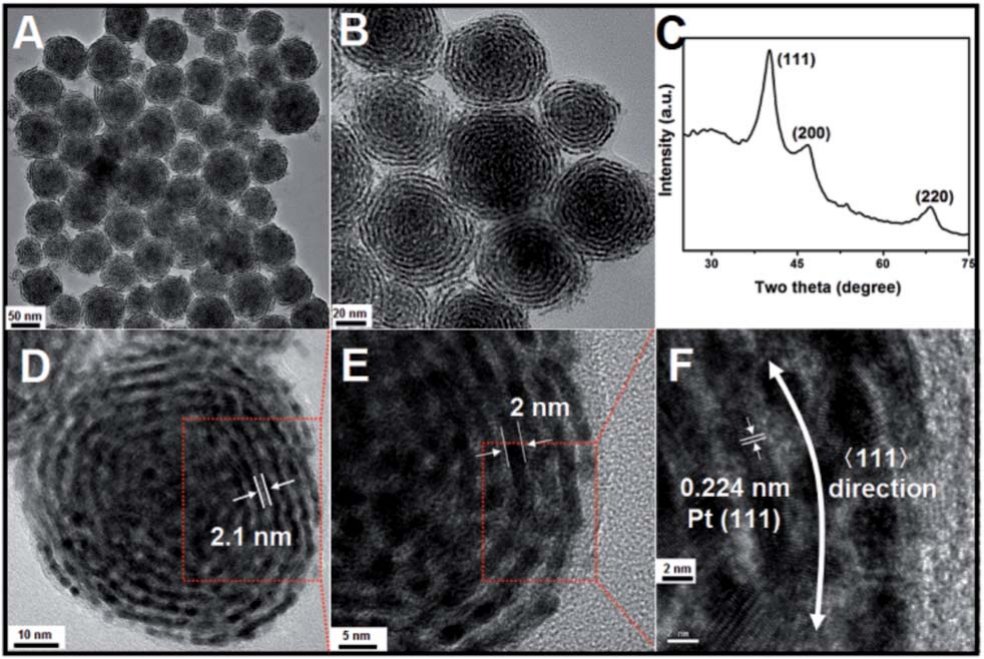
(A), (B) and (D–F): TEM images. (C) XRD data of the as-obtained Pt nanohelices.

The morphologies and structures of the as-obtained Pt products were investigated using different characterization techniques, including transmission electron microscopy (TEM), high-resolution transmission electron microscopy (HRTEM), and powder X-ray diffraction (XRD). As shown in Fig. 1A (a low-magnification TEM image), monodisperse, helix-like (here, the use of “helix” is to describe the curved nanostructures with ordered transverse channels) nanostructures with diameters of approximately 60 nm were obtained. Scattered, smaller-sized Pt nanoparticles could not be found. Interestingly, from the enlarged TEM image in Fig. 1B, we can see that the product exhibits a helical structure. The black threads can be attributed to the Pt crystals, and importantly, the helices have uniform pitches (the white areas, approximately 2.1 nm), as shown in Fig. 1D. By further enlarging the TEM images, it is observed that the nanohelices are composed of ultra-thin Pt nanowires (approximately 2 nm, Fig. 1E) coiled around a central point. In Fig. 1F, the HRTEM image shows that the lattice spacing (0.224 nm) corresponds well with the characteristic (111) planes of the Pt crystals, indicating that the as-prepared nanowires grow along the , the HRTEM image shows that the lattice spacing (0.224 nm) corresponds well with the characteristic (111) planes of the Pt crystals, indicating that the as-prepared nanowires grow along the 〈111〉 direction. The XRD analysis of the Pt nanohelical sample is shown in 111, the HRTEM image shows that the lattice spacing (0.224 nm) corresponds well with the characteristic (111) planes of the Pt crystals, indicating that the as-prepared nanowires grow along the 〈111〉 direction. The XRD analysis of the Pt nanohelical sample is shown in direction. The XRD analysis of the Pt nanohelical sample is shown in Fig. 1C. The peaks at 2*θ* = 40.1°, 46.7° and 68.1° can be indexed as the (111), (200) and (220) reflections of Pt, respectively. Impurity peaks are not seen in the XRD pattern. Noticeably, the diffraction peaks are broadened, indicating the presence of nanoscale structural features. The X-ray photoelectron spectroscopy (XPS) curves (Fig. S1†) identify two peaks at 71 eV and 74 eV that correspond well to the Pt 4f7/2 and 4f5/2 spin orbit peaks of metallic Pt, respectively.

**Fig. 2:**
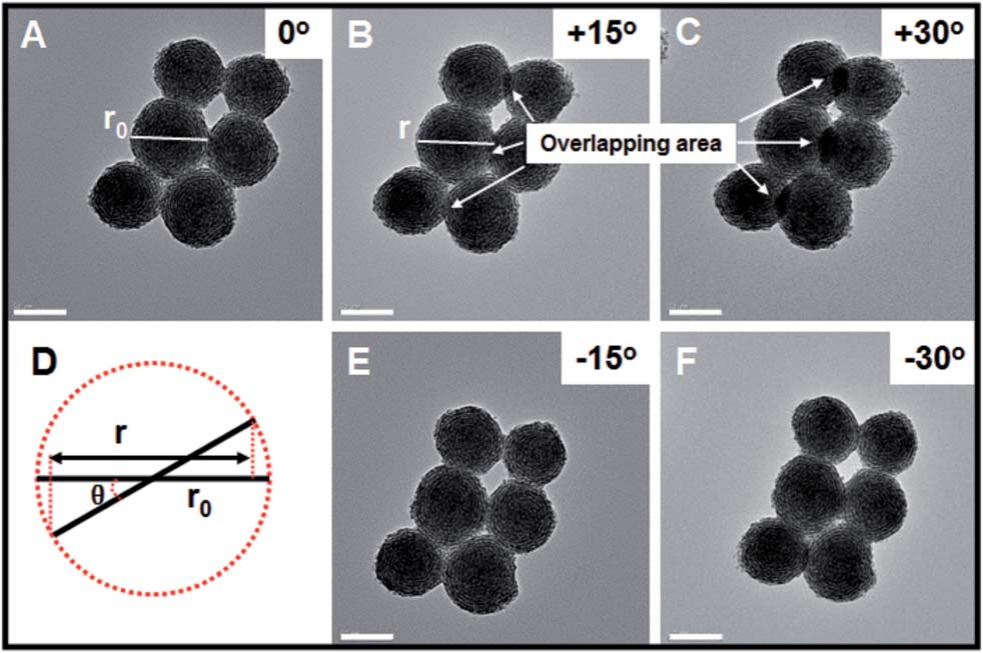
TEM images of the Pt nanohelices with different angles of rotation. The scale bar is 50 nm.

In order to further investigate whether the as-obtained Pt nanohelices take the form of three- or two-dimensional nanostructures, further TEM images, after *in situ* spinning of the zone axis, were obtained and are shown in Fig. 2. When the zone axis was spun to +30°, the overlapping areas became larger and larger (marked by the white arrows), which is direct evidence for successful spinning. If the as-obtained Pt nanohelices are two-dimensional, according to the Pythagorean theorem (as shown in Fig. 2D), the particle diameters should decrease to *r* (*r* = *r*_0_ × cos *θ*, where *r*_0_ is the original particle diameter and *θ* is the angle of rotation). After careful calculation, the difference in particle diameters between the un-rotated and rotated ones is negligible. A similar phenomenon is also observed when the zone axis is rotated to –30°. Thus, combined with the above results, this can confirm that the Pt nanohelices take the form of a three-dimensional sphere-like nanostructure.

**Fig. 3:**
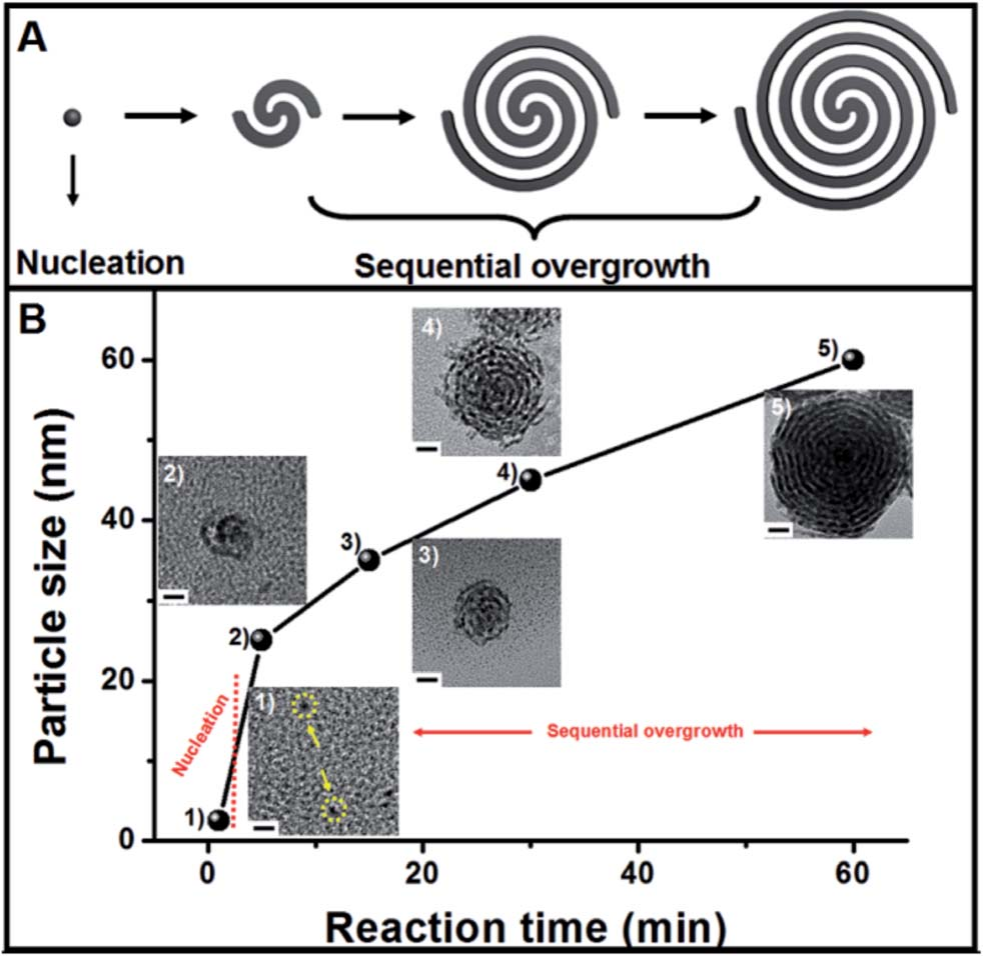
(A) Formation mechanism of the Pt nanohelices. (B) Change (with TEM images) in the diameter of overall nanohelices during the reaction: (1) 1 min, (2) 5 min, (3) 15 min, (4) 30 min, (5) 60 min (the scale bar in B is 10 nm).

The growth mechanism of the helical superstructures has been proposed using time-evolution TEM images. The superstructure formation can be broken down into two stages: nucleation and sequential overgrowth. As shown in Fig. 3B, after the addition of the AA solution at a reaction time of 1 minute, a few Pt nanoparticles (less than 2 nm) were found (Fig. 3B-1). After 5 minutes of reaction time, the original sub-15 nm Pt nanohelices were observed. The TEM image in Fig. 3B-2 shows that two Pt nanowires were coiled together, confirming the sequential overgrowth phenomenon. The nanohelices grew in size with increasing reaction time. The sizes of the nanohelices were 35 nm, 45 nm and 60 nm at 15 min, 30 min and 60 min, respectively. According to the TEM results, the growth mechanism can be understood as follows. An ion-exchange reaction first occurs between OTAB–Na and Pt^4+^ to form stable OTAB–Pt complexes (the OTAB–Pt complex can be easily separated from the growth solution as shown in Fig. S7†). The addition of AA triggers a redox reaction. However, the OTAB–Pt complexes significantly slow down the reaction rate. At the beginning of the reaction, only a few Pt seeds exist for the next growth process. The OTAB molecules also act as shape-directing agents to induce the overgrowth of Pt nanowires along the (111) direction through the strong interactions in the OTAB–Pt complexes until all of the OTAB–Pt complexes are consumed. As a result, uniform and monodisperse Pt nanohelices are successfully fabricated. The whole growth process is described in Fig. 3A.

Interestingly, the reaction is highly sensitive to the temperature. As shown in Fig. S2,† when the reaction temperature was increased to 80 °C, uniform sphere-like nanostructures were obtained. Moreover, the high-magnification TEM image in Fig. S2B,† shows the building blocks were different from those for the nanohelices. Instead of the original nanowires, Pt nanoparticles were observed. When the reaction temperature was further increased to 96 °C (with the data shown in Fig. S3†), ordered 3D nanostructures were not observed and only Pt nanoparticles were seen in the TEM images. This phenomenon is primarily caused by the fast redox reaction rate at higher temperatures. The increased reaction rate could cause two obvious changes. First, more Pt seeds are formed even at the beginning of the reaction, reducing the amount of OTAB–Pt in the solution; this environment would not favor sequential overgrowth. Second, when the reaction temperature is increased to 96 °C, homogeneous nucleation occurs, and the overgrowth process is skipped. Such growth phenomena are consistent with the growth mechanism described above.

**Fig. 4:**
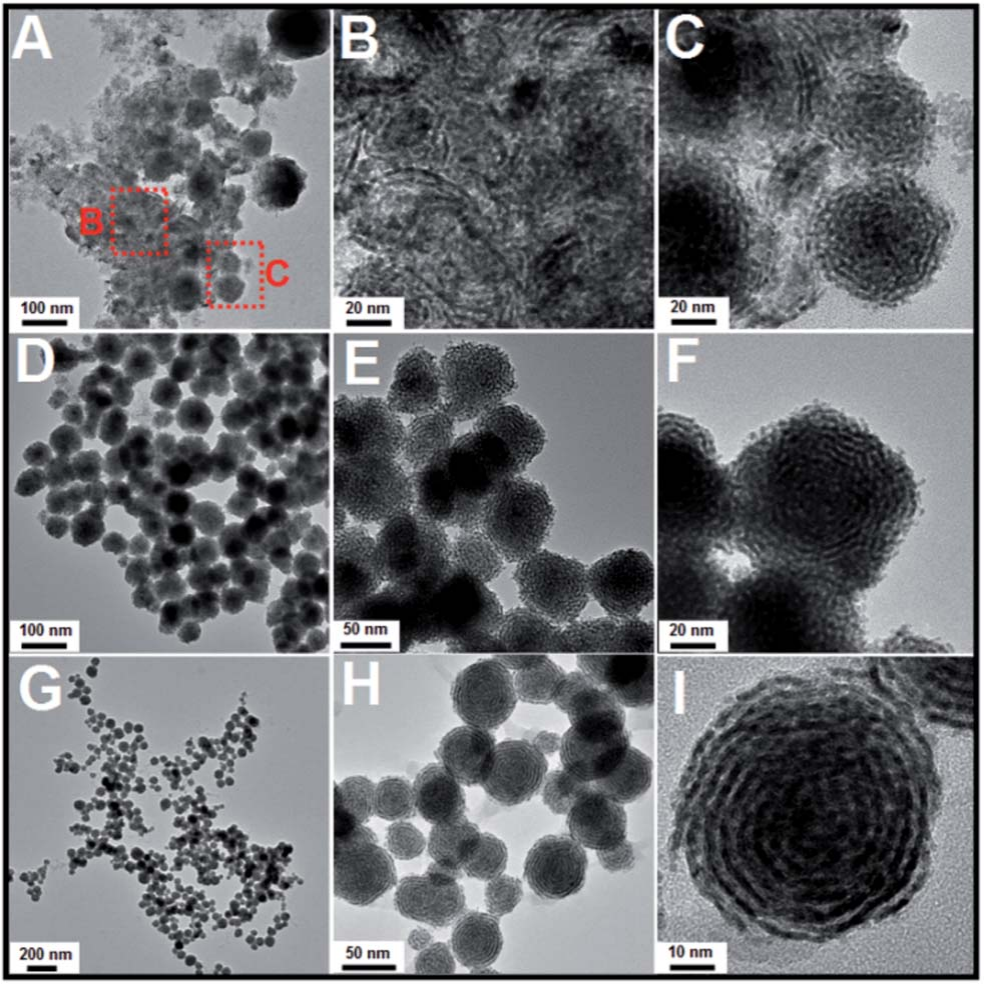
TEM images of Pt nanostructures obtained *via* the addition of different amounts of OTAB: (A) to (C): 20 mg; (D) to (F): 45 mg; (G) to (I): 135 mg (reactions at 60 °C).

Subsequently, a series of controlled experiments were performed to understand the effects of the amount of OTAB fed into the reaction solution on the formation of the Pt nanostructures. As shown in Fig. 4A to C, when 20 mg of OTAB was added to the reaction, some nanowires and assembled nanospheres were obtained. When the amount of added OTAB was increased to 45 mg, poor quality Pt nanohelices were observed (Fig. 4D to F), and many of these structures were broken, indicating that the overgrowth was less controlled. As shown in Fig. 4G to I, when 135 mg of OTAB was added, Pt nanohelices with large size distributions were obtained. These results illustrate that OTAB is key to the formation of the helical nanostructures. If the quantity of OTAB is insufficient, it cannot efficiently limit the independent nucleation of Pt; excess OTAB slows down the overgrowth process, causing large size distributions. We also investigated the role of the added AA in the reaction. As shown in Fig. S4 and S5,† changes in the amount of AA added did not affect the morphological evolution of the nanostructures.

Compared with previously reported Pt porous or branched nanostructures, the as-prepared Pt nanohelices have unique structural features, such as ultra-thin Pt nanowires as the building blocks instead of Pt nanoparticles, ordered and horizontal pore channels and highly curved helical nanostructures. Therefore, such nanostructures could provide channels for the rapid transmission of heat and electrons, and the thick, ordered, horizontal pore channels could largely increase the contact probability between a substrate and the Pt surface. Once substrates are spread in the pore channels, they are trapped, causing repeated impacts until the catalytic reactions are complete. Furthermore, their refraction of light could be enhanced compared to other Pt nanostructures.

**Fig. 5:**
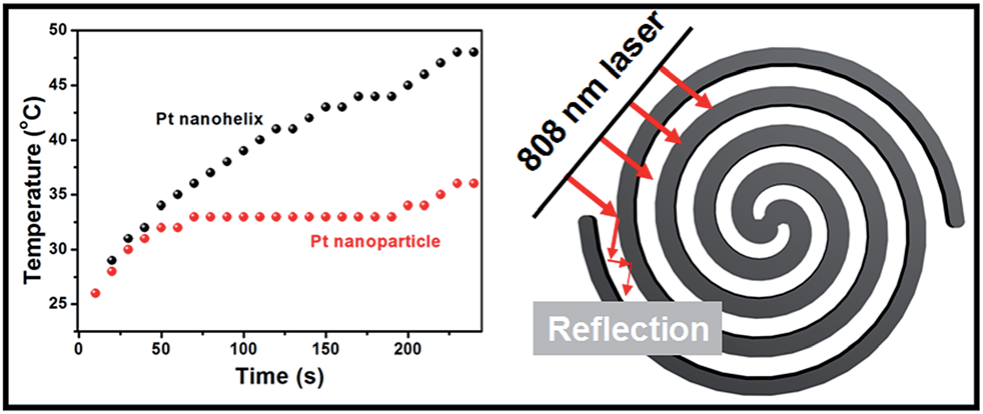
Photothermal tests on the Pt nanohelices and 3 nm Pt nanoparticles.

Inspired by these helical nanostructures with open-ended channels, we further studied the photothermal properties of the as-obtained Pt nanohelices. Under 808 nm laser irradiation, a continuous and rapid increase in the temperature of the Pt nanohelical solution occurs. As shown in Fig. 5, four minutes later, the solution reached a temperature of approximately 48 °C. The temperature of the Pt nanohelices increased by 22 °C (from room temperature), while the temperature of Pt nanoparticles only increased by 10 °C under the same reaction parameters. The NIR photothermal conversion efficiency of the Pt nanohelices is more than twice that of the Pt nanoparticles. Furthermore, UV-Vis spectra of the Pt nanohelices and nanoparticles have also been taken and are shown in Fig. S8.† Obviously, the Pt helices show a stronger visible-light absorption capability compared with the Pt nanoparticles. Thus, the main reasons for the highly enhanced photothermal properties of the Pt nanohelices could be attributed to the following two parts: (1) the strong absorption capability in the visible-light area and (2) the helical structures having unique reflection and absorption abilities, which has been observed in the CuS superstructures reported by Hu's group.^41^

The chemical reduction of 4-nitrophenol (4-NP) by ammonia borane (AB, NH_3_BH_3_) was chosen as a model reaction to evaluate the catalytic performances of the as-obtained Pt nanohelices and the classical porous Pt nanoparticles reported by Yang's group.^34^ AB is different from traditional hydrogen sources such as H_2_ and NaBH_4_ because it is much safer to use, non-toxic, and it has a high hydrogen capacity and good stability. As shown in Fig. S9,† compared with the traditional porous Pt nanoparticles, the as-obtained Pt nanohelices exhibited enhanced catalytic activity. The main reason could be attributed to the unique curved nanostructures which endow the nanowires with larger amounts of corner- and step-nanostructures compared with common nanowires.

## Conclusions

In conclusion, by controlling the reduction rate of the OTAB–Pt complexes and with the help of OTAB as a structure directing agent, Pt nanohelices with highly ordered horizontal pore channels were fabricated for the first time. Ultra-thin Pt nanowires (sub-2 nm) are the building blocks of these structures; they coil around a central point, leaving a sub-2.1 nm space between every two threads. In addition, the growth mechanism was studied in detail. The as-obtained helical nanostructures exhibit enhanced photothermal properties. The NIR photothermal conversion efficiency of the Pt nanohelices is more than twice that of the Pt nanoparticles. Their unique three-dimensional nanostructure has been considered as the main reason for this. It is believed that this unique helix-like nanostructure and the novel synthetic strategies have opened new windows for the design of highly active functional nanomaterials for real-world applications.

## Supplementary Material

Supplementary information
