# Supplementary material for: Pt nanohelices with highly ordered horizontal pore channels as enhanced photothermal materials
Source: Chem Sci. 2015 Jul 23;6(11):6420–4. doi: 10.1039/c5sc01686j (PMC5507185; doi:10.1039/c5sc01686j)
Supplement: Supplementary file 1 [file SC-006-C5SC01686J-s001.pdf]

Electronic Supplementary Material (ESI) for Chemical Science.
This journal is © The Royal Society of Chemistry 2015

Electronic Supplementary Information

Pt Nanohelices with Highly Ordered Horizontal Pore Channels as Enhanced Photothermal Materials

Shuyan Song,[a] Xiao Wang,[a] Sheling Li,[b] Zhuo Wang,[a] Qi Zhu,[b] and Hongjie Zhang[a,]*

a. *State Key Laboratory of Rare Earth Resource Utilization, Changchun Institute of Applied Chemistry, Chinese Academy of Sciences, Changchun 130022, P. R. China.*

b. *School of Material Science and Engineering, Changchun University of Science and Technology, Changchun, 130022, P. R. China.*

E-mail: hongjie@ciac.ac.cn

Experimental Section:

***N,N*-dimethyloctadecylammonium bromide acetate sodium salt ($C_{18}H_{37}$-$N(CH_3)_2(CH_2COONa)\cdot Br$, OTAB-Na):** the synthetic process is much similar to our previous report,[s1, s2] where just sodium bromoacetate instead of zinc bromoacetate was used. Typically, *N,N*-dimethyloctadecylamine (Aldrich; 10 mmol) and sodium bromoacetate (5 mmol) were dissolved in absolute ethanol (50 mL) with vigorously stirring and refluxed for 24 h in a 100-mL round-bottomed flask. The solution was then concentrated on a rotary evaporator to about 4 mL. This solution was cooled to room temperature and anhydrous diethyl ether (20 mL) was added to precipitate OTAB-Na as a white solid, which was isolated by filtration and washed with anhydrous diethyl ether.

Pt nanohelices: 90 mg of OTAB-Na and 10 mL of water were mixed together and heated at 60 ^oC, until a clear, transparent solution was obtained. Then 1.25 mL of H_2PtCl_6 (20 mM) aqueous solution was added in the reaction solution followed by injection of 1 mL freshly prepared aqueous ascorbic acid solution (0.1 M). Two hours later, the mixture was cooled down to room temperature and collected by centrifugation with the help of ethanol and washed three times with water and ethanol.

Pt nanoparticles: the synthetic process is adapted from our previous report.[s3] Typically, 0.1 mmol of H_2PtCl_6 was mixed with 0.5 mmol of PVP in the presence of 15 mL ethylene glycol and 5 mL of water, followed by ultrasound treatment for 10 minutes by using an ultrasonic cell crusher. The as-obtained products were purified by acetone, washed with water for three times.

Porous Pt nanoparticles: the synthetic process ismuch similar to the previous reports.[s4, s5] typically, the mixture of CTAB (0.1 M, 5.0 mL) and K_2PtCl_4 (0.01 M, 2.0 mL) was heated to 70 ^oC for10 min until the solution became clear. Ascorbic acid (0.02 M, 3.0 mL) was then added. The resultant mixture was kept at 70^oC for 8 h.

Characterization: The X-ray diffraction patterns of the products were collected on a Rigaku-D/max 2500 V X-ray diffractometer with Cu-Kα radiation (λ = 1.5418 Å), with an operation voltage and current maintained at 40 kV and 40 mA. Transmission electron microscopic (TEM) images were obtained with a TECNAI G2 high-resolution transmission electron microscope operating at 200 kV. XPS measurement was performed on an ESCALAB-MKII 250 photoelectron spectrometer (VG Co.) with Al Kα X-ray radiation as the X-ray source for excitation.

For temperature measurement due to the photothermalconversion on as-synthesized Pt nanohelices material: 808 nm NIR laser lightwas delivered through a quartz cuvette containing water (0.5 mL) and 50 uL Pt nanohelices or porous Pt DMF solution (0.005 M, determined by ICP). The light source was an external 0–0.3 W adjustable 808 nm semiconductor laser device. A thermocouple was inserted into the aqueous dispersion perpendicular to the path of the laser. The temperature was measure donce per 10 second.

Chemical Reduction of 4-NP by AB: The catalytic test was carried out in a quartz curette in the UV/vis/NIR spectrophotometer. The aqueous solutions of 4-NPions (0.01mmol/mL) and AB (0.2 mmol/mL), were freshly prepared. Typically 0.02 mL of nitrophenoland 0.05 mL mL of AB aqueous solution were mixed in 3 mL of H_2O. Then 20 μL of catalysts (0.005 mmol/mL, calculated form Pt, determined by ICP) were quickly added. The intensity of the absorption peak at 400 nmfor 4-NP ions was monitored byUV−vis spectroscopy along with time.

[s1] D. P. Liu, G. D. Li, Y. Su, J. S. Chen,*Angew. Chem. Int. Ed.* 2006, **45**, 7370.

[s2] Y. Zhang, D. P. Liu, X. Wang, S. Y. Song, H. J. Zhang, *Chem. Eur. J.* 2011, **3**, 920.

[s3] X. Wang, D. P. Liu, S, Y. Song, H. Zhang, *Chem. Comm.* 2012, **48**, 10207.

[s4] C. Fang, H. Jia, S. Chang, Q. Ruan, P. Wang, T. Chen, J. Wang, *Energy Environ. Sci.* 2014, **7**, 3431.

[s5] H. Lee, S. E. Habas, S. Kweskin, D. Butcher, G. A. Somorjai, P. D. Yang, *Angew.Chem., Int. Ed.* 2006, **45**, 7824.

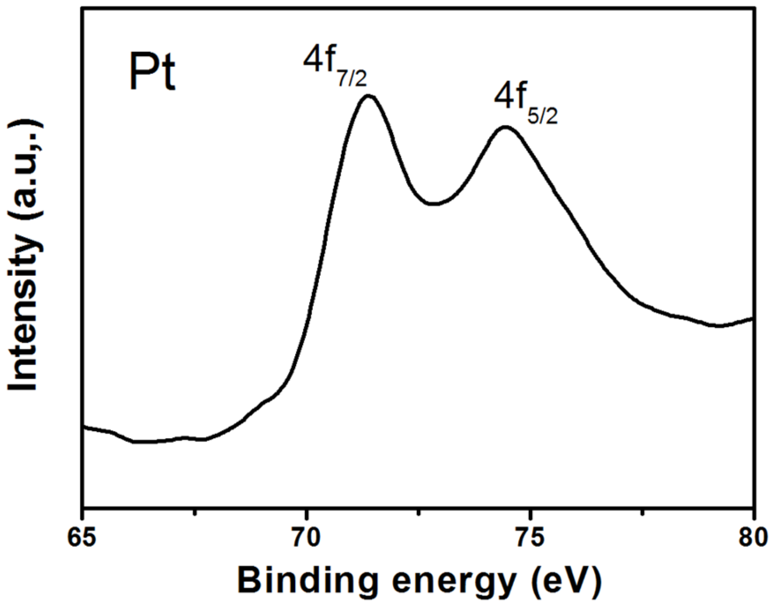

Fig. S1. XPS spectrum of Pt nanohelices.

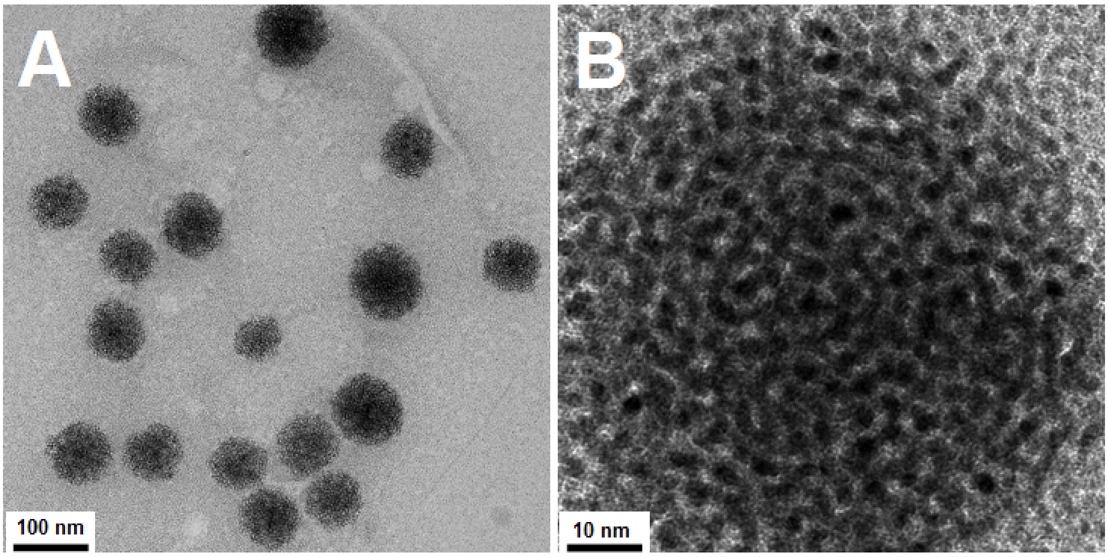

Fig. S2. TEM images of Pt porous nanospheres obtained at 80 ^{o}C.

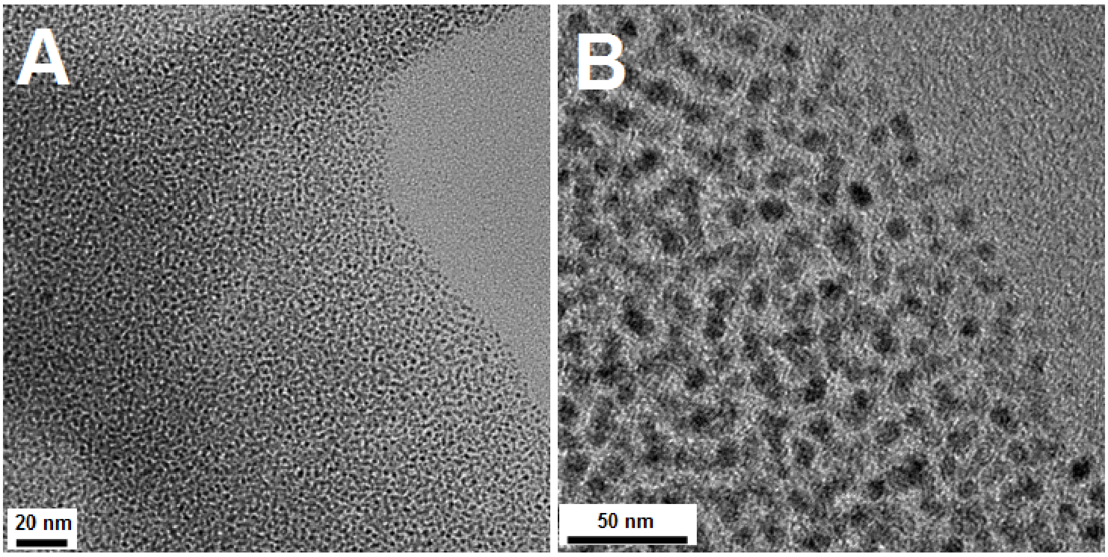

Fig. S3. TEM images of Pt nanoparticles obtained at 96 ^{o}C.

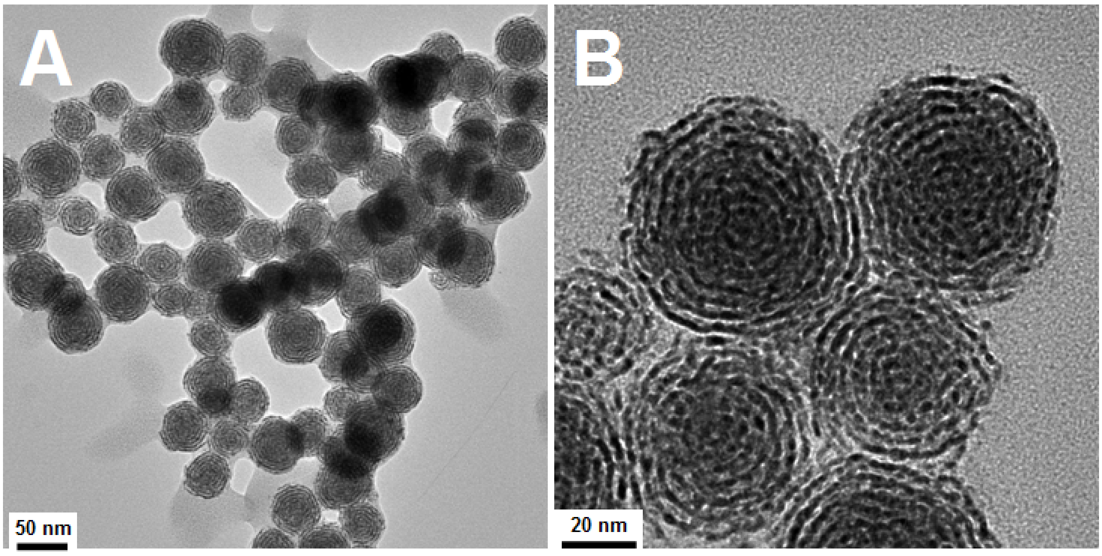

Fig. S4. TEM images of Pt nanohelices obtained by addition of 0.5 mL AA (0.1 M).

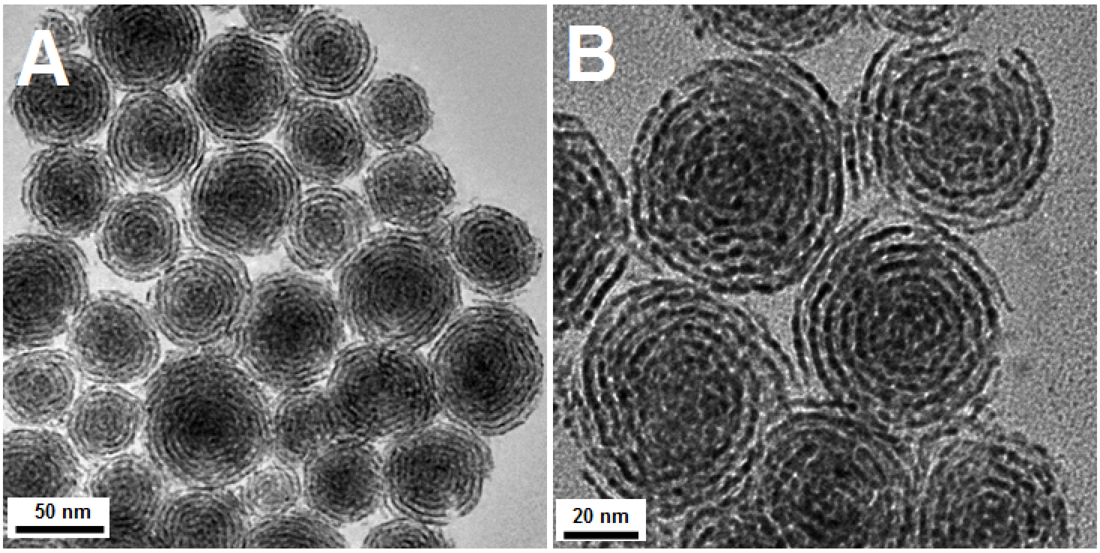

Fig. S5. TEM images of Pt nanohelices obtained by addition of 3 mL AA (0.1 M).

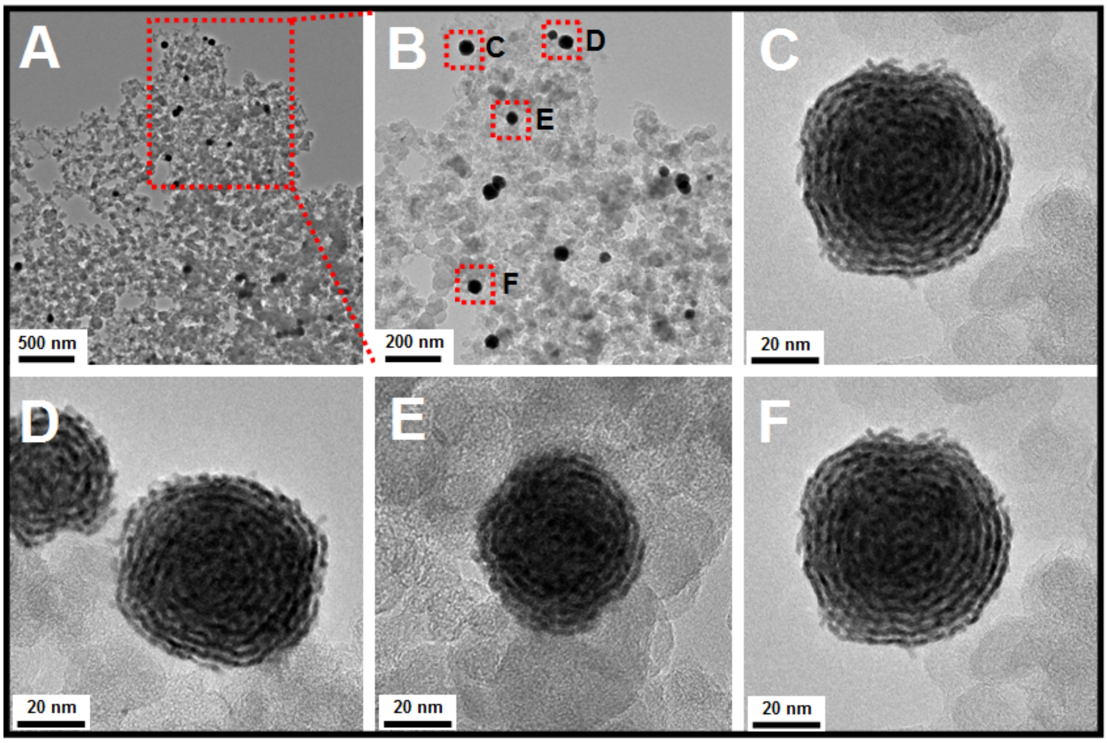

Fig. S6. TEM images of Pt nanohelices supported on XC-72R.

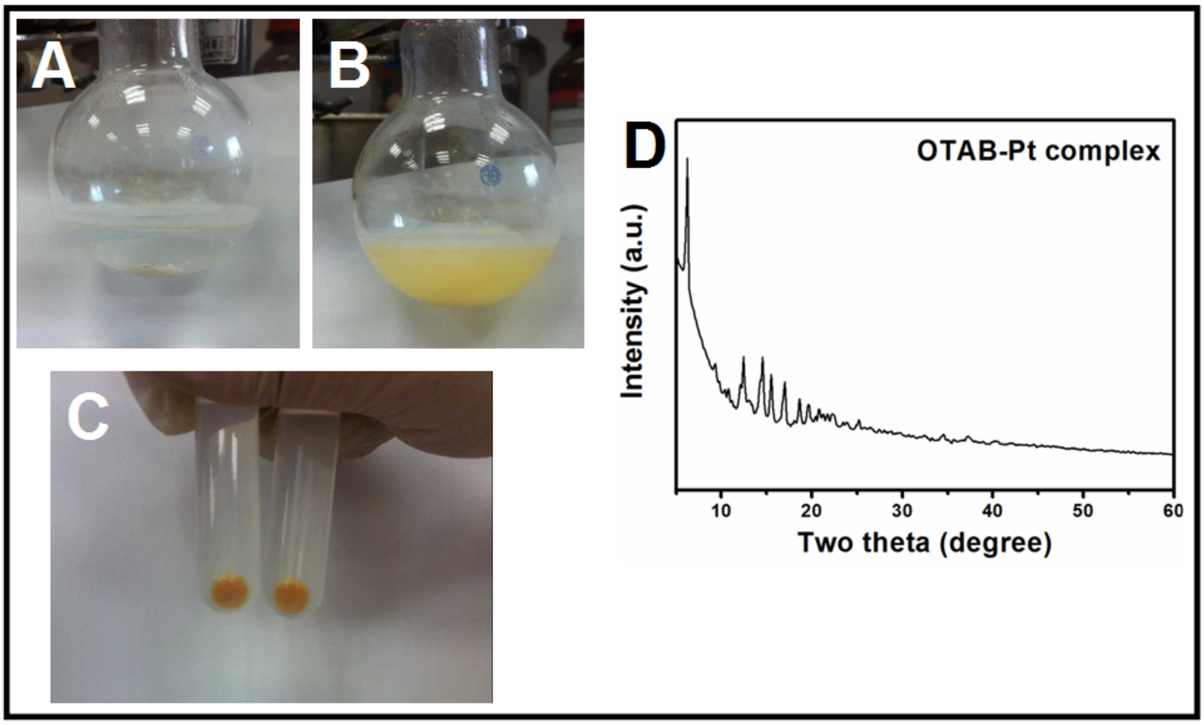

Fig. S7. Photos of (A): OTAB-Na aqueous solution (60 ^{o}C); (B) after addition of H_2PtCl_6 aqueous solution; (C) purified OTAB-Pt complex by centrifugation at 6000 rpm for 2 min. (D) XRD pattern of OTAB-Pt complex.

After the addition of H_2PtCl_6 in OTAB-Na aqueous solution, the original colorless and clear solution became turbid very soon. The as-obtained light yellow precipitations are easily purified by simple centrifugal separation without the addition of any anti-solvent. It is the first direct evidence of the formation of OTAB-Pt complex. Furthermore, XRD analyses have also been taken. The XRD spectrum has confirmed that the product is not metallic and be in the form of layered nanostructure (the similar XRD data has been illustrated in the Ref. S1). Combined with the above analyses, it could confirm that OTAB-Pt complex has been formed during the synthesis.

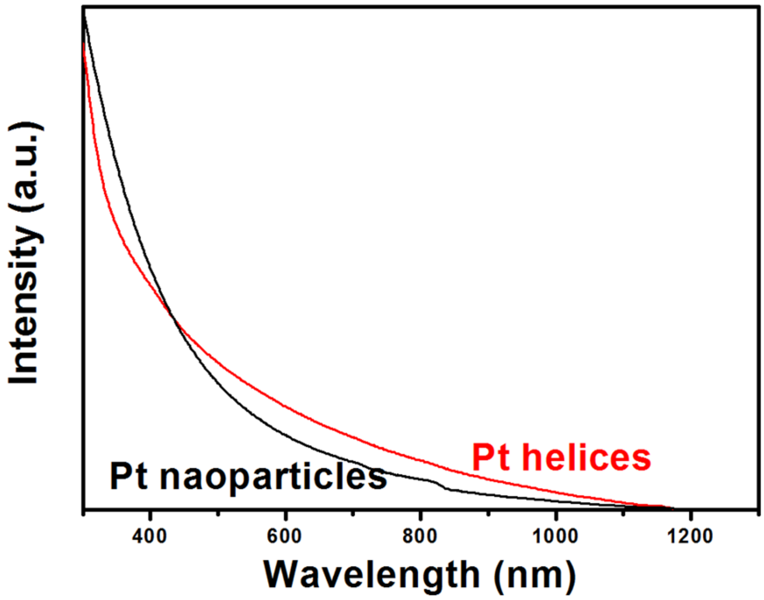

Fig. S8. UV-vis spectra of the Pt nanohelices and nanoparticles (with the same concentrations).

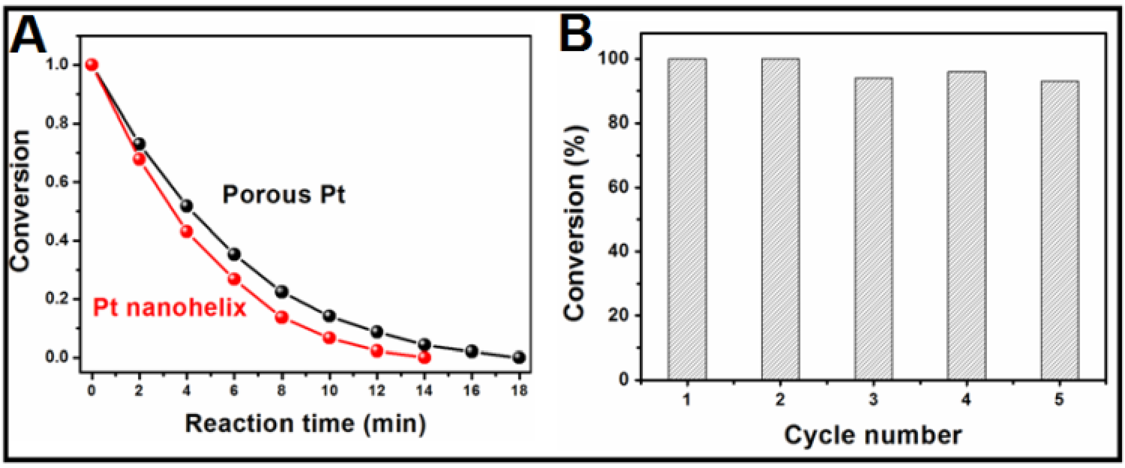

Figure S9. Catalytic tests of Pt nanohelices and porous Pt nanostructures. The chemical reduction of nitrophenol by ammonia borane was studied.

Before the catalytic property tests, both of the samples were loaded onto commercial carbon black, XC-72R, via a simple dipping method. A DMF solution containing Pt nanohelices was slowly added to a XC-72R-DMF mixture at 80 °C. Four hours later, the black products were collected at the bottom of the container. As shown in Fig. S6, the as-obtained Pt nanohelices were loaded onto the carbon surface uniformly. Scattered particles were not found outside of the boundary of the carbon supports, indicating a successful hybridization. More importantly, from the high-magnification TEM images in Fig. S6C to F, it was observed that the Pt nanohelices were stable. After the loading process, they maintained their morphologies.

It is well-known that the 4-NP solution exhibits a strong absorption peak at 317 nm under neutral and acidic conditions. As the alkalinity of the solution increases, 4-NP ions become the dominant species, producing a spectral shift to 400 nm. To simplify the catalytic test, a 4-NP solution was first reacted with NaOH (1:1) to completely form 4-NP ions. When a Pt nanohelix/C sample was added to the catalytic solution, many bubbles were generated in the cuvette. The initial catalytic reaction rate was very fast, and gradually slowed down. Meanwhile, the bright yellow solution faded completely and became colorless. As shown in Figure S9, after 14 min, the Pt nanohelix/C sample had catalyzed the 100 % reduction of 4-NP by AB. In contrast, porous Pt nanoparticle/C catalysts required approximately 18 minutes to accomplish the catalytic reaction. The turnover number (TON, at 50% conversion) values of two samples are also calculated as 0.29 min^{-1} and 0.23 min^{-1} for Pt helices and porous Pt nanoparticles, respectively. The highly increased catalytic performance of Pt nanohelix/C sample could be attributed to the unique cured nanostructures which makes the nanowires be with more numbers of corner- and step-nanostructures compared with common nanowires. The substrate-immobilized nanocatalysts can be easily recovered and recycled. As a reference, un-loaded Pt nanohelix has shown a better catalytic activity than loaded ones, however, the

sample is hard to be separated from the reaction solution owing to the strong “Brown motion” (with the data shown in Fig. S10). Thus, such experiment has confirmed that the hybridization of Pt nanohelix and carbon support is very necessary. A cycling test was done by performing the catalytic reaction five times. As demonstrated in Figure S9B, the reaction stopped immediately once the catalyst was removed and resumed again with similar kinetics after reintroduction of the catalyst. The performance stability of the nanocatalyst was further examined by repeatedly using the same catalyst. Between each cycle, the supported catalyst was removed from the reaction without washing it. After five successive cycles (reaction time of 30 min), over 93 % of the 4-NP was reduced, suggesting that the nanohelices have the promising catalytic stability.

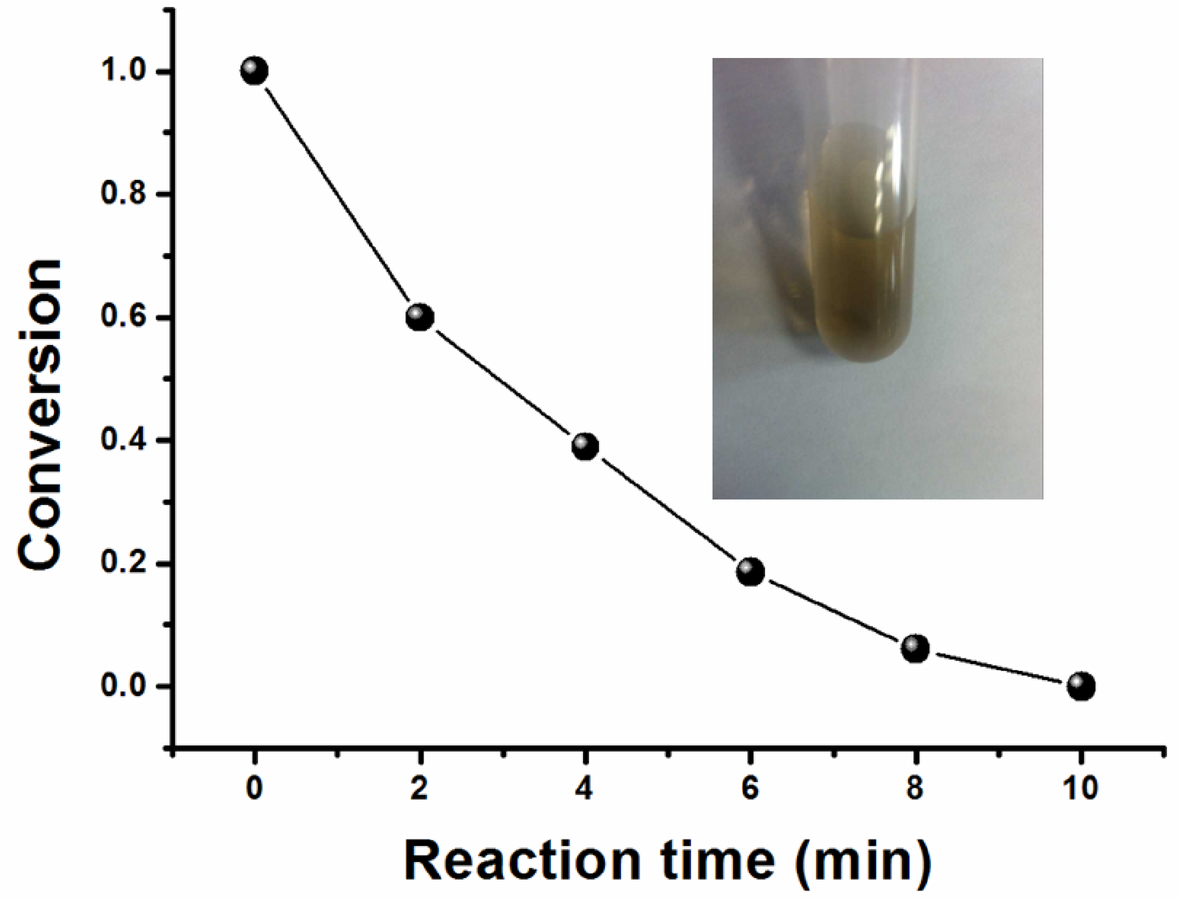

Fig. S9. Catalytic tests of Pt nanohelices without loading on carbon. The corresponding photo is the catalysts after centrifugation at 10000 rpm for 10 min.
